# PopP2 interacts with PAD4 in an acetyltransferase activity-dependent manner and affects plant immunity

**DOI:** 10.1080/15592324.2021.2017631

**Published:** 2022-01-03

**Authors:** Sung Un Huh

**Affiliations:** Department of Biology, Kunsan National University, Gunsan, Republic of Korea

**Keywords:** Lipase like protein, PAD4, EDS1, ralstonia effector, PopP2, acetylation

## Abstract

Plant pathogenic bacteria inject many of the effector proteins into host cell to manipulate host protein and promote pathogen development. Only a few effectors can be recognized by plant immune receptors called nucleotide‐binding domain and leucine‐rich repeat‐containing proteins (NLRs). Enhanced disease susceptibility1 (EDS1) is an important regulator of plant basal and NLR receptor-triggered immunity. EDS1/PAD4 or EDS1/SAG101 heterodimers are recruited by Toll-interleukin1-receptor domain NLRs (TNLs) to transcriptionally mobilize resistance pathways. Type III effector PopP2 contributes to *Ralstonia solanacearum* virulence. PopP2 has an acetyltransferase activity and is recognized by Arabidopsis NLR pair RPS4/RRS1-R. On the other hand, PopP2 avirulence function is dependent on its enzymatic activity but target proteins in the host cell are still largely unknown. In this study, we found EDS1 and PAD4 are new host targets of PopP2 effector. Arabidopsis PAD4 lipase-like domain protein physically associates with enzymatic active PopP2 protein but not inactive PopP2^C321A^. PAD4-PopP2 interaction is disrupted by EDS1 immune regulator but not SAG101. We propose that acetyltransferase activity of PopP2 might confer specificity to PAD4 to manipulate plant immunity. As a counter strategy, EDS1 associates with PAD4 to form heterodimeric immune regulator complexes for activating basal resistance and interfering PopP2 physical interaction.

Plant pathogens cause disease in crop plants and lead to a loss in food productions.^1^ To overcome plant disease, remarkable investigation has been achieved in our understanding of the molecular basis of plant disease defense mechanisms.^2–4^ For example, plant immunity induced by salicylic acid (SA) is vital for resistance to bacterial pathogens.^5^ SA accumulation is enhanced by Arabidopsis *enhanced disease susceptibility 1* (*EDS1*) with coregulator *phytoalexin deficient 4* (*PAD4*).^6^ EDS1/PAD4 protein complexes serve as important immune regulators in basal and receptor triggered immunity.^7^ EDS1 physically interacts with PAD4 and senescence-associated gene 101 (SAG101) which is identified as a senescence-associated gene. EDS1 can form a ternary complex with PAD4 and SAG101 to mediate plant defense signaling.^8^

*Ralstonia solanacearum* is a soil-borne bacterium causing bacterial wilt on many Solanaceous crops, such as potato, pepper and tomato.^9,10^ *R. solanacearum* encodes up to 80 putative type III secreted effectors (T3SEs).^11^ Among them, Pseudomonas outer protein P2 (PopP2) functions as a virulence factor through acetyltransferase activity.^12–15^ As a counter strategy, Arabidopsis NLR immune receptor *Resistant to R. solanacearum 1-R* (RRS1-R) protein can directly recognize PopP2 effector via acetylation of RRS1-R WRKY domain by PopP2.^13,15^

PopP2 belongs to the Yersinia outer protein J (YopJ) family that acts as an acetyltransferase on host targets to suppress plant immunity.^3^ *Salmonella enterica* AvrA and *Vibrio parahaemolyticus* VopA are members of the YopJ family. AvrA and VopA acetylate-specific serine, threonine, and lysine residues in mitogen-activated protein kinase kinases (MAPKKs) to suppress kinase enzymatic activation.^16,17^ HopZ4, a member of the YopJ family from the cucumber pathogen *Pseudomonas syringae* pv. *lachrymans* interacts with the proteasomal subunit RPT5 to inhibit proteasome activity during infection.^18^ *P. syringae* HopZ1a is a YopJ homologue and destroys plant microtubule networks with acetyltransferase activity.^19^ Since RRS1-R, Arabidopsis Cys protease Responsive to Dehydration 19 (RD19), and some of WRKY transcription factors physically interact with PopP2 but no other plant targets of PopP2 have yet been described.^14,15,20^

In previous studies, we found that EDS1 interactions with *P. syringae* effector AvrRps4 are disrupted by PAD4 *in vivo* suggesting that EDS1/PAD4 protein heteromeric complexes might interfere with the pathogen effector interaction.^21^ Interestingly, PopP2 and AvrRps4 have an identical target of the WRKY domain of RRS1-R immune receptor.^13^ These results suggest that PopP2 could be associated with the immune modulators EDS1 or PAD4. First, we examined whether PopP2 associates with PAD4 *in planta*. We expressed C-terminally HA-tagged PAD4 protein with 35S::GFP or 35S::PopP2-GFP or a nonfunctional with inactive catalytic mutant 35S::PopP2^C321A^-GFP in *N. benthamiana* leaves, Following co-immunoprecipitation (co-IP) with HA or GFP beads, active PopP2 only associates with PAD4 but not inactive PopP2^C321A^ protein (Figure 1a). To further investigate acetylation of PAD4, we used acetyl-lysine specific antibody to detect acetylation of PAD4. However, PAD4 exhibited no acetylation and autoacetylation is detected in PopP2 protein (Figure 1b). Similarly, we performed co-IP with EDS1 and PopP2. EDS1 interacts with both PopP2 and PopP2^C321A^ protein (Figure 2a) but PopP2/EDS1 interaction exhibited quite weak signals in coIP. EDS1 also shows no acetylation via acetyl-lysine specific antibody (Figure 2b). These data demonstrate that enzymatic active PopP2 specifically targets PAD4. EDS1 also associates with PopP2 independent of acetyltransferase enzymatic activity.

**Figure 1.**
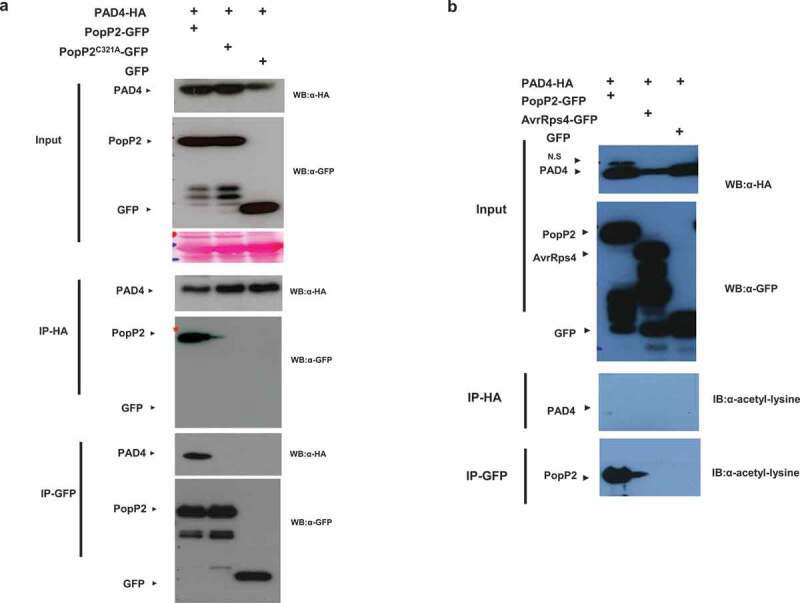
Enzymatic active PopP2 associates with PAD4 *in planta*. (a) Anti-HA immunoprecipitation of PopP2-GFP, PopP2^C321A^-GFP, and GFP in the presence of PAD4-HA. Samples were prepared from transiently co-expressed PopP2-GFP, PopP2^C321A^-GFP, and GFP in the presence of PAD4-HA in *N. benthamiana* leaves. Total extracts were immunoprecipitated with anti-HA or anti-GFP beads followed by immunoblotting with α-HA and α-GFP antibodies. (b) Anti-HA and -GFP immunoprecipitations of PopP2-GFP, AvrRps4-GFP, and GFP in the presence of PAD4-HA. Samples were prepared from transiently co-expressed PopP2-GFP, AvrRps4-GFP, and GFP in the presence of PAD4-HA in *N. benthamiana* leaves. After the cell extracts were immunoprecipitated with anti-HA or anti-GFP beads, acetylated proteins were detected using Ac-K antibody. AvrRps4 were used as a negative control.

**Figure 2.**
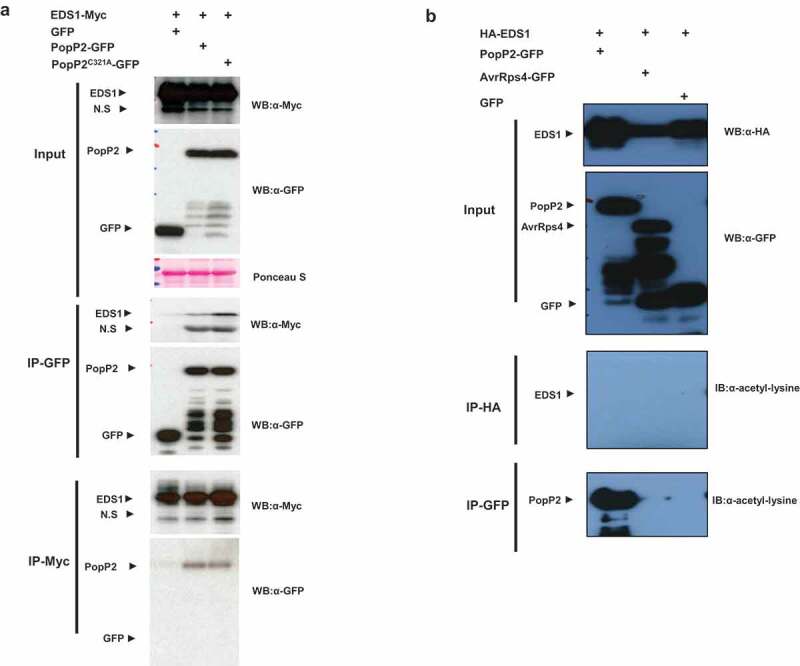
EDS1 physically interacts with PopP2 *in planta*. (a) Anti-GFP immunoprecipitation of PopP2-GFP, PopP2^C321A^-GFP, and GFP in the presence of EDS1-Myc. Samples were prepared from transiently co-expressed PopP2-GFP, PopP2^C321A^-GFP, and GFP in the presence of EDS1-Myc in *N. benthamiana* leaves. Total extracts were immunoprecipitated with anti-GFP or anti-Myc beads followed by immunoblotting with the α-Myc and α-GFP antibodies. (b) Anti-HA and -GFP immunoprecipitations of PopP2-GFP, AvrRps4-GFP, and GFP in the presence of HA-EDS1. Samples were prepared from transiently co-expressed PopP2-GFP, AvrRps4-GFP, and GFP in the presence of HA-EDS1 in *N. benthamiana* leaves. After the cell extracts were immunoprecipitated with anti-HA or anti-GFP beads, acetylated proteins were detected using Ac-K antibody. AvrRps4 were used as a negative control.

Next, we investigated whether EDS1 can disrupt PopP2/PAD4 associations. As shown in Figure 3a, PAD4 coIPs with PopP2 but not PopP2^C321A^ protein. PAD4/PopP2 associations are disrupted by co-expression of EDS1 protein. To confirm this result, we used bimolecular fluorescence complementation (BiFC) assay. As expected, very weak BiFC signals between PopP2^C321A^-cCFP and nVenus-PAD4 were observed in the nucleus (Figure 3b). A strong BiFC signal between PopP2-cCFP and nVenus-PAD4 was observed in the nucleus but not co-expressed EDS1-Myc protein (Figure 3b), suggesting that formation of EDS1/PAD4 heterodimeric complexes might attenuate PopP2/PAD4 association.

**Figure 3.**
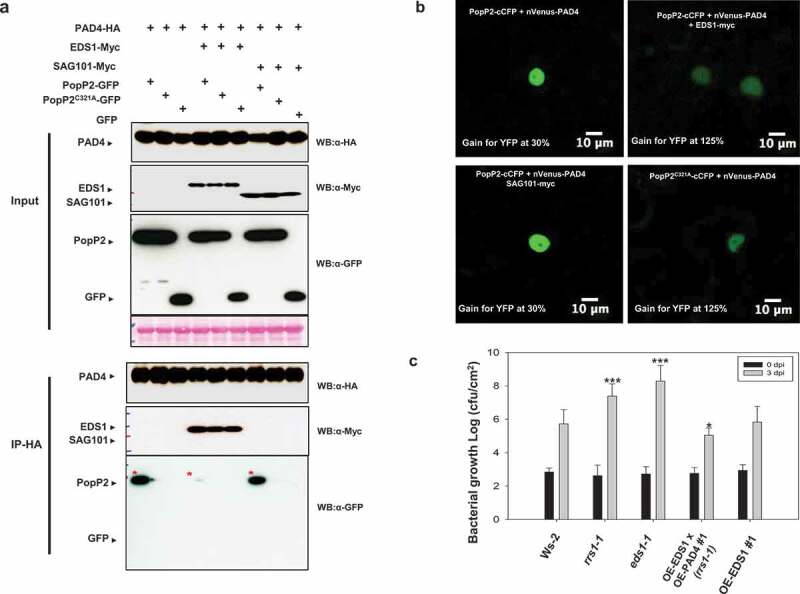
PAD4/PopP2 interaction is attenuated by EDS1 co-expression but not SAG101 *in planta*. (a) PAD4-HA or PAD4-HA/EDS1-Myc or PAD4-HA/SAG101-Myc were transiently co-expressed with PopP2-GFP, PopP2^C321A^-GFP or GFP in *N. benthamiana* leaves. Immunoprecipitations were performed using anti-HA agarose beads and then analyzed by immunoblot with the α-Myc, α-HA, and α-GFP antibodies. (b) BiFC analysis reveals that PAD4/PopP2 association is strongly reduced in the presence of EDS1 but not of SAG101. Co-expression of PopP2-cCFP with nVenus-PAD4 shows strong YFP signals that are strongly reduced in the presence of EDS1-Myc but not in the presence of SAG101-Myc. Co-expression of PopP2^C321A^-cCFP with nVenus-PAD4 shows very weak YFP signals. BiFC assays were performed by co-expression of the indicated proteins in *N. benthamiana*. Images were obtained at 2 days post-infection (dpi). The experiment was repeated three times with similar results. (c) Bacterial growth assay using *P. syringae* pv. *tomato* (*Pto*) DC3000-delivered AvrRps4N:PopP2^149−488^ system in Ws-2, *rrs1-1, eds1-1*, 35S::EDS1 (*eds1-1* mutant background), and 35S::EDS1 x 35S::PAD4 (*rrs1-1* mutant background) transgenic Arabidopsis plants. Leaves of four-weeks-old Arabidopsis plants were syringe-infiltrated with *Pto* DC3000 strains and bacterial growth was determined at 3 dpi. Error bars indicate standard deviations (n = 5). Student’s t-test; *P < .01, ***P < .0001.

We also tested the effect of PAD4 and SAG101 on PopP2 association using co-IP and BiFC assays in *N. benthamiana* leaves. In co-IP, PAD4 shows no association with SAG101 because of the absence of EDS1 (Figure 3a). PAD4/PopP2 interaction was not significantly altered by SAG101 co-expression both co-IP and BiFC (Figure 3a,b). These results indicated that EDS1 specifically inhibited PAD4/PopP2 association when transiently co-expressed in *N. benthamiana*.

To determine the effect of disruption of PopP2 interaction, we tested bacterial growth using *P. syringae* pv. *tomato* (*Pto*) DC3000-delivered AvrRps4N:PopP2^149−488^ system^22^ in Ws-2, *rrs1-1, eds1-1*, and 35S::EDS1 x 35S::PAD4 (*rrs1-1* background) transgenic Arabidopsis plants. As expected, Ws-2 Arabidopsis plants contained RRS1-R immune receptor exhibited strong effector triggered immunity but not *rrs1-1* (Figure 3c). Mutant *eds1-1* showed enhanced susceptibility to bacteria although RRS1-R still recognized PopP2. Overexpressing transgenic plants of PAD4/EDS1 in *rrs1-1* showed strong basal defense (Figure 3c), suggesting that formation of EDS1/PAD4 dimeric complexes might have a role in inhibition of PopP2 interaction to enhanced plant immunity.

In conclusion, this study provides evidence that PopP2 effector targets both immune regulator EDS1 and PAD4 to suppress plant immunity. Basically, immune regulator EDS1/PAD4 heterodimeric complex can elevate plant basal defense signaling. Moreover, *Phytophthora capsici* effector PcAvh103 interacts with EDS1 to disrupt EDS1/PAD4 and inhibit plant defense signaling, suggesting that EDS1 and PAD4 might be general effector targets.^21,23^ On the other hand, EDS1 disrupts PAD4/PopP2 interaction but not SAG101. This result consistent with disruption of EDS1/AvrRps4 interaction by PAD4.^21^ Notably, we found PAD4 only associates with enzymatic active PopP2. It is possible that acetylation activity of PopP2 might modified PAD4 protein to enhance protein interaction affinity. We failed detection of acetylation in EDS1 and PAD4 using acetyl-lysine specific antibody and it might be required more specific method applications. Further studies are still needed to explore the PAD4 acetylation by PopP2 to understand host target modification. We therefore propose that PopP2 acetyltransferase activity may be required to specific interaction with host target proteins to suppress plant immunity. As a counter strategy, plant EDS1 makes heterodimeric immune regulator complexes with PAD4 for activating basal resistance and interfering PopP2 physical interaction.
